# SEED: A novel web-based data visualization platform to visualize, communicate, and explore social, environmental, and equity drivers of health

**DOI:** 10.1017/cts.2024.569

**Published:** 2024-09-16

**Authors:** Nrupen A. Bhavsar, Jessica Sperling, Raquel Ruiz, Dinushika Mohottige, Perusi Muhigaba, Mina Silberberg, Anthony Leiro, Pamela Maxson, Michelle Lyn, L. Ebony Boulware

**Affiliations:** 1 Department of Surgery, Division of Surgical Sciences, Department of Biostatistics and Bioinformatics, Duke University School of Medicine, Durham, NC, USA; 2 Social Science Research Institute, Duke University, Durham, NC, USA; 3 Clinical and Translational Sciences Institute, Duke University School of Medicine, Durham, NC, USA; 4 Department of Medicine, Icahn School of Medicine at Mount Sinai, New York, NY, USA; 5 Department of Family Medicine and Community Health, Duke University School of Medicine, Durham, NC, USA; 6 Division of Community Health, Duke University School of Medicine, Durham, NC, USA; 7 Department of Medicine, Duke University School of Medicine, Wake Forest School of Medicine, Winston-Salem, NC, USA

**Keywords:** Social determinants of health, health equity, community engagement, electronic health records, health disparities, data visualization

## Abstract

Multisector stakeholders, including, community-based organizations, health systems, researchers, policymakers, and commerce, increasingly seek to address health inequities that persist due to structural racism. They require accessible tools to visualize and quantify the prevalence of social drivers of health (SDOH) and correlate them with health to facilitate dialog and action. We developed and deployed a web-based data visualization platform to make health and SDOH data available to the community. We conducted interviews and focus groups among end users of the platform to establish needs and desired platform functionality. The platform displays curated SDOH and de-identified and aggregated local electronic health record data. The resulting Social, Environmental, and Equity Drivers (SEED) Health Atlas integrates SDOH data across multiple constructs, including socioeconomic status, environmental pollution, and built environment. Aggregated health prevalence data on multiple conditions can be visualized in interactive maps. Data can be visualized and downloaded without coding knowledge. Visualizations facilitate an understanding of community health priorities and local health inequities. SEED could facilitate future discussions on improving community health and health equity. SEED provides a promising tool that members of the community and researchers may use in their efforts to improve health equity.

## Background and significance

It is well known that the neighborhood within which a person lives can greatly impact their health. Concentrated poverty, violence, lack of access to healthy food and green space, limited educational opportunities, and poor air quality are associated with adverse health outcomes. The inequitable distribution of these social drivers of health (SDOH) is linked to health inequities [1].

Developing interventions to address the root causes of these health inequities requires multi-stakeholder engagement by members of the community in tandem with healthcare providers. Intervention development also requires these groups to easily access tools that quantify and characterize the distribution of SDOH and its associations with health outcomes.

Historically, SDOH data have been in disparate data sources and have been challenging to link. Even when these data have been compiled to inform clinical care or health policy, the results of these studies are often disseminated through traditional academic sources, such as academic journals or policy white papers [2]. Often these sources are not available to individual community members or those working outside of traditional research enterprises.

Novel, democratized (i.e., accessible) tools that help stakeholders visualize and quantify SDOH and their association with health outcomes may have multiple key benefits, particularly if this information is available to stakeholders from varied arenas including community-based and grassroots organizations, healthcare delivery systems, public health entities, research organization, government, and commerce. These tools can expand the evidence base for stakeholders seeking to establish associations between SDOH and health outcomes, and they may also enable users to identify areas of greatest need so that resources can be distributed more equitably, and policies can be tailored to address key SDOH barriers to health. Democratized data visualization platforms can further empower communities to promote community-engaged and informed approaches to address local health concerns by facilitating partnerships between community members health systems and researchers [3].

However, in their raw format, SDOH and health data are not easily available or comprehensible to the lay public. Even when data visualization tools are implemented, they are often developed for experienced users such as health system administrators, clinicians, and/or researchers and are not designed for community members. We describe our development and implementation of a web-based data visualization platform designed to (1) help community users visualize the prevalence of social and environmental conditions within a neighborhood and (2) expose real-world data from an academic medical center and a federally qualified health center (FQHC) to communicate the prevalence of health conditions within community neighborhoods. The platform, called the Social, Environmental, and Equity Drivers (SEED) Health Atlas, aims to facilitate better awareness and dialog on community health needs and to bolster efforts to improve health equity.

## Methods

### Overview

**Figure 1.**
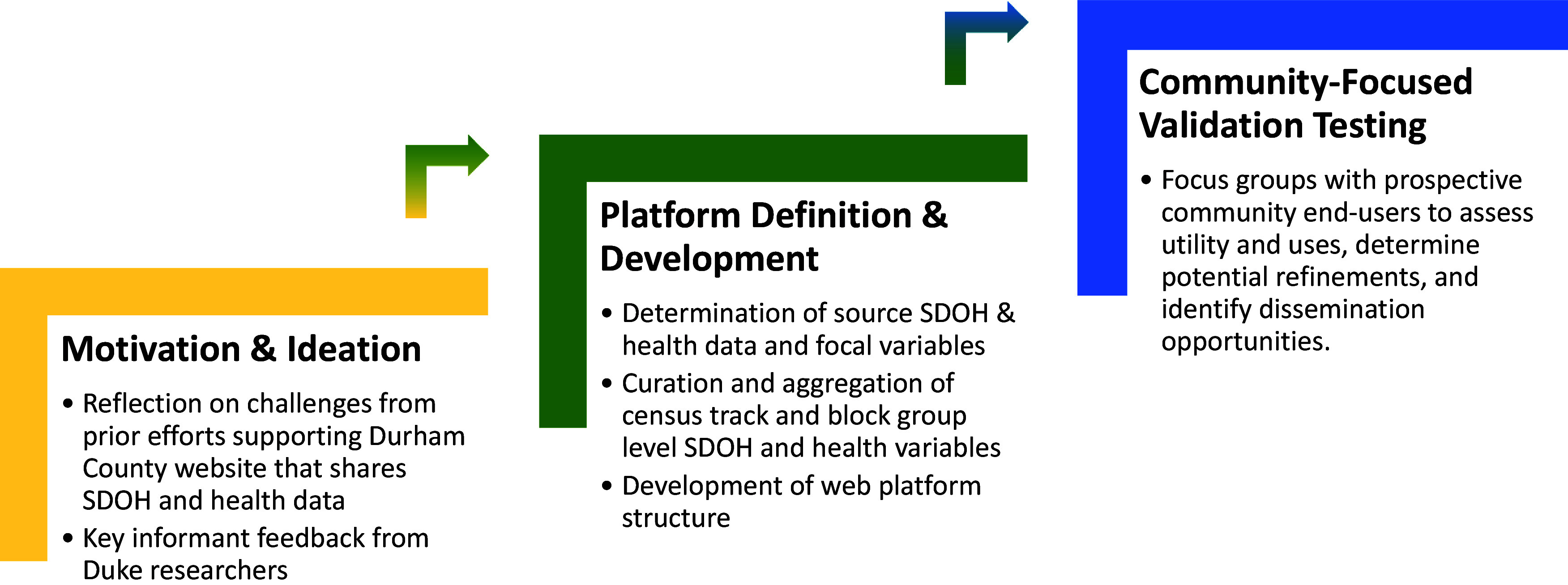
Social, Environmental, and Equity Drivers (SEED) Health Atlas development process. SDOH = social determinants of health.

We developed and deployed a web-based data visualization platform to make SDOH and residential health data visible and available to the community. We conducted key informant interviews and focus groups with leaders and representatives of potential end-user populations, including community members, public health officials, and researchers, to establish web platform user needs and desired functionality (Figure 1). SDOH and de-identified aggregated electronic health record (EHR) data were shared on the platform using tested visualizations.

### Platform development

#### Motivation

Development of SEED was motivated by our prior experience supporting a local, Durham County-specific website that shared SDOH and health data. Through this process, we recognized the need to expand the geographic coverage, technical capabilities, and SDOH and health data presented through a novel web platform. We also elicited key informant feedback from Duke researchers on available digital tools that could address these needs and aspects of a novel tool that would be beneficial when engaging with the community. We conducted key informant interviews with six potential end users across Duke University faculty whose research incorporated SDOH, including faculty with expertise in economics, environmental health, statistics, sociology, law, and medicine. Faculty were prompted with questions pertaining to what data they used, where did they obtain the data, and how they were used.

### Curating data

#### SDOH data

We developed an initial list of SDOH variables to include on the platform by considering the five domains defined by the US Department of Health and Human Services, Office of Disease Prevention and Health Promotion in Healthy People 2030 [4]. These include economic stability, education access and quality, healthcare access and quality, neighborhood and built environment, and social and community context. Within these domains, we chose variables that have been commonly used in published research and are available. This list included data on socioeconomic factors, environmental pollutants, built environment, deprivation indices, and health outcomes (Table 1). Sources of these data included but were not limited to federal (e.g., US Census, Environmental Protection Agency [EPA], the Centers for Disease Control and Prevention [CDC]) and local (Durham County) agencies. Neighborhood resources and aspects of the built environment were obtained from a third-party dataset that included information on businesses within the USA. This information was summarized as a number of businesses (e.g., bars/nightclubs) per census tract or block group.

**Table 1.** Social determinants of health (SDOH) constructs and data elements available on Social, Environmental, and Equity Drivers (SEED)

SDOH construct	Data element	Source
Economy	Below poverty	ACS
	Median household income	ACS
	Owned at least one vehicle	ACS
	Unemployed	ACS
Education	Bachelor’s degree or higher	ACS
	High school or less	ACS
Environment	PM 2.5 (fine particulate matter)	NATA
	Acetaldehyde	NATA
	Benzene	NATA
	Carbon tetrachloride	NATA
	Diesel PM	NATA
	Ethylbenzene	NATA
	Formaldehyde	NATA
	1,3-Butadiene	NATA
	Hexane	NATA
	Lead compounds	NATA
	Manganese compounds	NATA
	Mercury compounds	NATA
	Methanol	NATA
	Methyl chloride	NATA
	Nickel compounds	NATA
	Toluene	NATA
	Xylenes	NATA
Food access	Low access tract at 1 mile for urban areas and 10 miles for rural areas	USDA
	Low access tract at 1 mile for urban areas and 20 miles for rural areas	USDA
	Low access tract at 1/2 mile for urban areas and 10 miles for rural areas	USDA
	Low access tract using vehicle access and at 20 miles in rural areas	USDA
	Low income and low access tract measured at 1 mile for urban areas and 10 miles for rural areas	USDA
	Low income and low access tract measured at 1 mile for urban areas and 20 miles for rural areas	USDA
	Low income and low access tract measured at 1/2 mile for urban areas and 10 miles for rural areas	USDA
	Low income and low access tract using vehicle access or low income and low access tract measured at 20 miles	USDA
Housing	Cost-burdened mortgage holders	ACS
	Cost-burdened renters	ACS
	Evictions	Eviction Lab
	Eviction filing rate	Eviction Lab
	Eviction filing	Eviction Lab
	Eviction rate	Eviction Lab
	Median gross rent	ACS
	Median home value	ACS
	Out of state ownership	ACS
	More than one person per room	ACS
Indices	Area Deprivation Index	University of Wisconsin
	Socioeconomic Status Index Score	AHRQ
	Child Opportunity Index	diversitydatakids.org
	Social Vulnerability Index	CDC
Infrastructure and amenities	Bar or nightclub	InfoUSA
	Impervious area	Durham Compass
	Liquor stores	InfoUSA
	Parks	InfoUSA
	Religious locations	InfoUSA
	Tobacco retail outlets	Primary data collection
	Tree coverage	Durham Compass
	Walkability	EPA
	Broadband	FCC

ACS = American Community Survey; SDOH = social determinants of health; NATA = National Air Toxics Assessment; USDA = United States Department of Agriculture; AHRQ = Agency for Healthcare Research and Quality; CDC = Centers for Disease Control and Prevention; EPA = Environmental Protection Agency; FCC = Federal Communications Commission.

#### Community health data

Health data were obtained from the EHR of the Duke University Health System and Lincoln Community Health Center (LCHC), the only FQHC in Durham County, NC, where Duke is located. Individuals receiving care at the LCHC and Duke University Health System (DUHS) share a common patient identifier as they are geographically close and use the same EHR vendor. These data represent at least one encounter at LCHC or DUHS by 85% of Durham County residents [1]. Health conditions of interest included chronic kidney disease, diabetes, hypertension, low birth weight, myocardial infarction, stroke, and overweight/obesity (Supplemental Table 1). The conditions were phenotyped based on established definitions [5–7] and in consultation with clinicians utilizing diagnosis code, laboratory values, and other clinical indicators. Prevalence estimates for each condition were summarized at the census tract and block group level. The process to aggregate individual-level data at the census block group level addressed concerns to maintain privacy and to ensure nondisclosure of protected health information. As part of this process, a third-party honest broker determined the minimum number of events within a block group and census that would be permissible to report and visualize.

#### Developing a prototype

We considered a list of features (e.g., categories of SDOH data, types of visualization, download function) that could facilitate a conversation on the social and environmental factors that impact health. We worked with web-based data visualization platform developers and used this set of features to create wireframe mockups of the web-based data visualization platform and iterated with our development team to design the layout of the platform, categories for the variables, and technical features that would facilitate use of the SDOH and health data. After multiple rounds of wireframe development, a prototype web-based data visualization platform was created.

#### Community end-user engagement

After the initial prototype was created, we examined the end-user perspective with a focus on relevance for community groups. We specifically focused on community groups given likelihood of lesser data familiarity, compared to researcher end users. That is, we wanted to determine if the website prototype was appropriate for those with lesser data familiarity. We solicited feedback from community research liaisons within the Duke Clinical and Translational Science Institute (CTSI), who routinely serve as liaisons between community members and community-based organizations within Durham County; researchers, a multidisciplinary advisory group of Duke University faculty and staff with expertise in sociology, epidemiology, and health equity; and members of the Durham County Department of Public Health.

This initial perspective informed the development focus of two subsequent formal focus groups with potential end users from the community, specifically leaders and representatives of community-based organizations dealing with health and SDOH issues (e.g., food access). Participants were solicited during the Durham Community Health Summit where the CTSI Community Engaged Research Initiative (CERI) had a table promoting their program with the community. The CERI staff asked individuals if they would be interested in providing feedback on SEED. A total of 12 contacts resulted from this recruitment (7 community members, 5 community leaders), and an email invitation was sent to these contacts to participate or asking them to refer another member of their organization in April 2023. Two focus groups (*n* = 10 total participants across both) occurred via Zoom in April and May 2023. In the sessions, participants were first provided with a demonstration of SEED wherein they could also briefly ask NAB questions about the platform. JS then led a semi-structured discussion addressing aspects of the value/utility, user experience, and community outreach/dissemination mechanisms. Following data collection, data were reviewed for key themes using rapid response analysis [8]. In particular, JS and PM established a rapid analysis format for synthesizing themes, with initial structure aligning with main topics/issues addressed in data collection (e.g., value, ease of use). PM reviewed data recordings, transcripts, and notes taken during the focus groups to identify initial results within these areas, and JS and PM discussed emerging results to identify any opportunities for adjustment or clarification. While there was evidence of saturation on high-level themes (e.g., value of tool), data collection did not necessarily reach data saturation across all subareas (e.g., all specific potential reasons for value) [9]. This is in line with the exploratory nature of this phase and suggests that further community work with SEED could identify additional use value or desired capacities.

## Results

### Availability of existing resources

Key informant interviews with Duke faculty indicated that there was not a central resource with necessary data, which was accessible to researchers and community members with pertinent functionality. They found current websites that contain SDOH data challenging to use and reported that an easier interface would be helpful. They wanted a novel platform that collated the most used SDOH constructs. Many users did not have a programming background and were unable to link different SDOH data. They suggested it would be helpful if there was a way to link datasets without the need to code. This could be useful to community members as well. They also saw a need to communicate these data to the lay public. Respondents indicated they most often use census data, environmental pollutant data, and health data in their research. Results received from other potential end users within the Duke community and Durham Department of Health were similar on all key points.

### SEED atlas content and structure

#### SDOH data

The SEED web-based data visualization platform includes multiple SDOH constructs that broadly align with many of the Healthy People 2030 SDOH domains, such as economics, education, healthcare access, neighborhood and built environment, and social and community context (Table 1). Census data included categories such as income, education, and housing-related variables. Environmental pollutant data included air pollution as defined by pollutant size (e.g., particulate matter 2.5 [PM_2.5]_) and other pollutants from the toxic release inventory.

#### Health data

**Figure 2.**
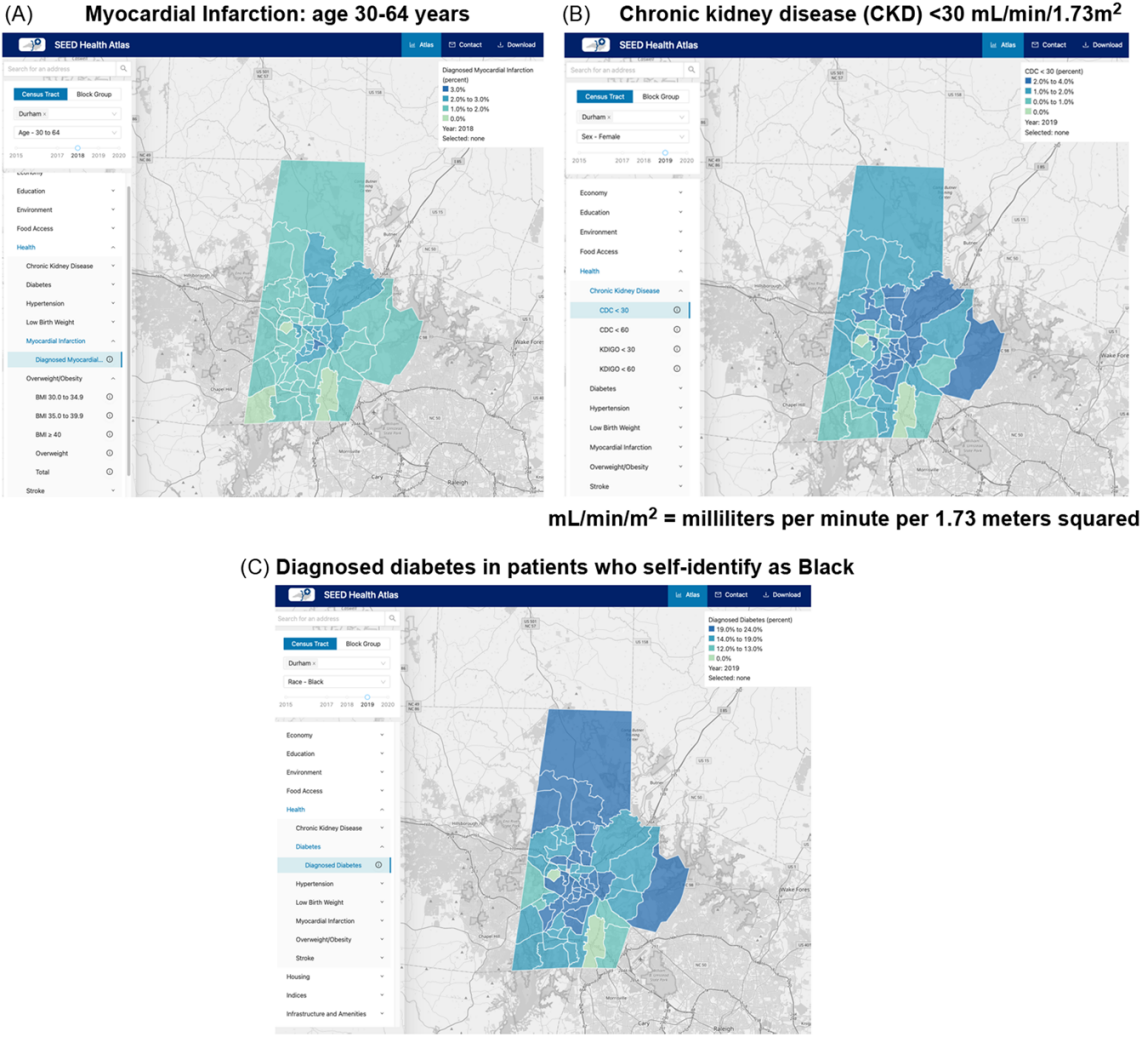
Health data stratified by age, sex, and race/ethnicity on the Social, Environmental, and Equity Drivers (SEED) Health Atlas.

The web-based data visualization platform displays de-identified, summarized data from patients who received care at DUHS or LCHC on multiple health conditions, including acute (e.g., myocardial infarction) and chronic (e.g., diabetes) conditions, and those that are impacted more immediately (e.g., overweight/obesity) and distally (e.g., chronic kidney disease). These data can be viewed by age, race/ethnicity, and sex subgroups (Figures 2a–c).

### Technical elements

#### Geographic levels and scope

**Figure 3.**
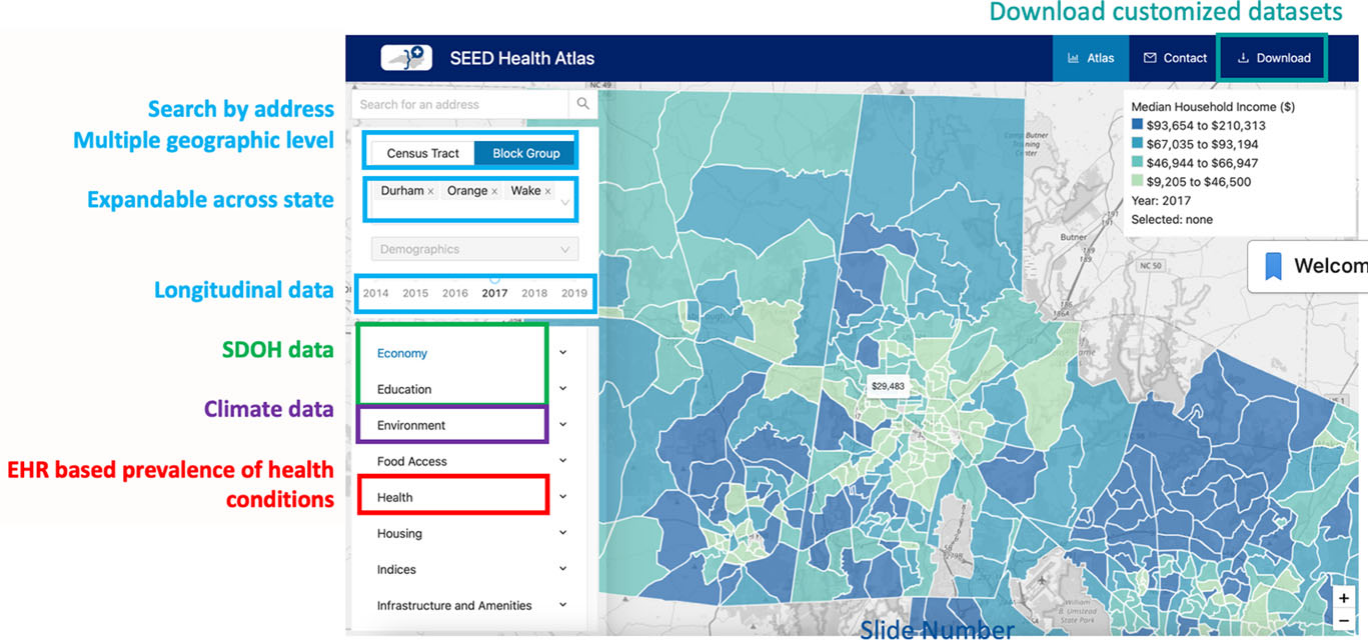
Technical features of the Social, Environmental, and Equity Drivers (SEED) Health Atlas. SDOH = social determinants of health; EHR = electronic health records.

There are many technical features on the platform to facilitate use across various users (Figure 3). The platform allows data to be visualized at multiple geographic levels, including census tract and block group. This is important as different SDOH can be available at different geographic levels (e.g., Area Deprivation Index vs. Social Vulnerability Index). Most individuals do not know the census tract or block group within which they live. To aid rapid identification, the platform has a search function that allows users to enter their address and identify the census tract or block group within which they live. The platform also includes a map base layer for the entire USA, allowing for layers of data to be mapped across local, regional, and national geographies. Data currently on the platform are mapped to counties that are proximal to Durham County, NC, with plans to expand regionally and nationally.

#### Data types

The platform can display multiple data and file types. These include multiple validated indices of deprivation, including the Area Deprivation Index (ADI), the Social Vulnerability Index (SVI), and the Child Opportunity Index. There are also numerous individual variables available on the platform. These include data on socioeconomic factors (e.g., poverty, education), the built environment (e.g., number of parks, liquor stores within a block group), and environmental pollutants (e.g., fine particulates [PM2.5]). For environmental pollutants such as air pollution, grid-defined data (e.g., 1 km x 1 km grid) are assigned to census tracts and/or block groups before posting on the platform. The platform also provides data on key infrastructure, such as neighborhood walkability and broadband access, which is an essential utility. Critically, many of these data are available longitudinally, with some available annually (e.g., census data), while other data are available every few years (e.g., ADI, SVI). SEED provides a data dictionary that explains how SDOH and health data are defined, promoting transparency and reproducibility.

#### Data democratization

A key functionality on the platform is the ability to visualize and share data with different end users. All data on the platform can be visualized at multiple geographies, longitudinally, and, for health data, by specific subgroups such as age and race. In addition, customized datasets can be created on the platform without the need to write code. Data on SEED include a column for census tract and/or block group level FIPS codes. Within DUHS, and common to other health systems, patient addresses are also geocoded to FIPS codes at the census tract and block group level through enterprise geocoding solutions. This allows the lay public and researchers to download the data and link to other data sources, including EHR data.

### Community user feedback

Results from the focus groups show participants value the content and ease of use of the SEED platform while also suggesting domains where further information is desired. Participants also identified promising dissemination strategies.

#### Value and utility

Participants communicated that the SEED Health Atlas is useful because it made data about community characteristics, needs, and assets easily accessible. As one respondent explained:

*It’s information frankly that I didn’t know the public had access to, and it with, with information. You, you can gain a lot of knowledge, and looking at the data know what your community really needs, what’s lacking. And it’s a quick, easy way to go in, look at the subcategories, pick what you’re trying to focus on and get some real time relevant data.*

They appreciated the breadth of data available on the platform and noted how these data can be used, including for pursuing funding opportunities, engaging in community advocacy, and program planning. Participants highlighted the important role that data access plays in grant writing as grants increasingly request data, and it has taken a significant amount of time to locate data across sources when they previously applied for grants. One participant indicated that a web platform such as SEED would be helpful in advocating for changes within the Durham Public Schools, as they require quantitative data before action. The platform also allows users to examine trends over time and place, which participants reported could be helpful to understand the impact of interventions or related work. Participants indicated that the data were appropriately granular, as the platform allows one to view data at the census tract and block group level. Participants primarily reported that the SEED Health Atlas was easy to navigate, even on mobile devices: (“I thought it was very easy…I was looking specifically at the food insecurity, and I didn’t have any trouble maneuvering and seeing the different regions”).

#### Areas for development

While participants valued the breadth of data and indicated data elements and quality were sufficient for some domains, such as health and housing, they offered suggestions for adding data in other domains. This included foci such as education levels, neighborhood resources and community assets (food access/pantries, community economy information, minority-owned businesses, resources for people who do not speak English), measures of social isolation, and community opinion data (to assess community needs). Some respondents also encouraged specific community sites and site descriptions to be added, such as community organization demographics (e.g., church denomination and size). Regarding the presentation of data, users suggested adding a layering function that can assist in telling a compelling story about the data and potential relationships among factors (e.g., air pollution and health conditions). A participant noted that other geographies may be needed, such as school districts, as understanding neighborhoods served by different schools is not always done at the census tract level.

Participants were offered additional opportunities to improve user experience and use. They suggested explaining the purpose of the platform on the same webpage as the map itself and explaining the history of SEED Health Atlas. Platform users wanted additional information on data, including the source of data, how often these data were updated, and whether the updates were data automated. They recommended adding specific resources on how to use and share data from the platform, including guidance on how to cite the platform and its data (e.g., APA-formatted citations), examples of how the data had been used in the past, and simple analytic guidance for anyone accessing a full dataset (e.g., how to do comparisons). Participants indicated this type of guidance would assist users engaged in advocacy, as they would have a better idea of how to share what they have learned from the platform with decision-makers and make the data actionable. Finally, users recommended the platform’s content be translated into languages other than English. One participant said, “there’s people in the community that are doing amazing work, and some of them their English is limited, but as long as you give them the information…they’ll do miracles with it.”

#### Perspective on dissemination

Participants emphasized the importance of partnering with community members and organizations as a way to disseminate the SEED Health Atlas with the community, including meeting the community “where they are” and in ways they deem trustworthy. In addition, emphasis was placed on sharing the platform with community members in addition to community leaders (“It’s about reaching the people that are the pulse”). One participant clarified that sharing the SEED Health Atlas with community members, and not just community leaders, could evoke empowerment in community members to advocate for themselves through grassroots initiatives. Specific dissemination and outreach strategies include direct engagement in community events, visiting frequented community sites (e.g., churches, nail salons, barbershops), attending community organization meetings (e.g., coalition meetings), and sharing information with established community listservs. Participants also considered marketing other community entity resources on the SEED Health Atlas to create partnerships. Some participants addressed dissemination methods that may take the SEED Health Atlas beyond its original primary aim but could be valuable and considered – for instance, introducing the platform as an educational tool for students so that they could learn about data and their communities and gamifying the platform by having the public use it as an incentive to learn more about their neighborhood.

### Use case

The SEED Health Atlas’s potential to drive research and quality improvement projects within health systems has been evidenced via an initial use case focused on opportunities to address gun violence within areas served by the DUHS. This initiative utilized the SEED Health Atlas to develop a dashboard to identify patterns of gun violence [10]. The dashboard linked SDOH data from the platform with demographic and clinical information on gun violence victims from the Duke Trauma Registry and EHR. Insights from the dashboard, such as areas with greater social need and gun violence, were used to inform potential interventions and where to implement them. These include the Hospital-based Violence Intervention Program (an intensive case management intervention that provides “wraparound” services to gun violence victims and their families) and enrolling a small group of at-risk community members in GED classes to combat limited educational and career opportunities. Similar initiatives can be implemented at single or multiple health systems by scaling the platform. While this use case represents a health system effort, prior-described feedback from end users within the community highlighted the opportunity to use the platform to advocate for changes within community institutions (e.g., Durham Public Schools) and promote conversations with political leaders to improve neighborhoods.

## Discussion

Developing interventions to address health inequities requires contributions across multiple sectors. Accessible information is needed on SDOH that impacts health and health disparities. The SEED Health Atlas has been developed to provide multiple end users with a tool to identify neighborhood-level SDOH that impact health disparities. Platform development was informed by feedback from community members and researchers and includes the ability to visualize and download data without the need to code. Community members and other stakeholders can identify aspects of the neighborhood in which they live that inform health, and translational researchers can link data on the platform with EHR or other SDOH data to promote research and quality improvement initiatives. The use of SEED can facilitate discussions on community health and opportunities to reduce health inequities.

There were important lessons learned from the development of SEED that may be beneficial for others aiming to create similar tools. Curating and communicating accurate health and socio-contextual data require subject matter expertise. As an example, there are over 70 tables for personal income through 5-year range American Community Survey data. The SEED development team was multidisciplinary with expertise across social epidemiology, biostatistics, informatics, community engagement, data visualization, and web development. These individuals were skilled in identifying and summarizing SDOH constructs with applicability to the local context, geospatial statistics, geocoding and data linkage, data visualization and communication, and platform programming. It was equally critical to have buy-in from DUHS and the FQHC to provide linked EHR data, with bidirectional communication and goals for population health improvement between community stakeholders and health system partners. There are understandable concerns about patient confidentiality and data governance, which also offer important lessons for others developing these platforms. When summarizing de-identified EHR data, it was helpful to work with a third-party honest broker to define the minimum number of people within a census tract or block group for whom we could display and democratize health data for common chronic conditions. SEED can serve as a model to other localities seeking to communicate or democratize similar information, which can be leveraged to improve population health and SDOH. SDOH data are readily available across the USA. Accurately defining the prevalence of health conditions can be challenging if data are not available from the health systems from which a majority of residents receive care.

There are ongoing efforts by others to democratize SDOH and clinical data through websites [2]. Health departments [11,12], academic centers [13], and government organizations [14–16] have created websites to disseminate this information. However, not all of these websites include comprehensive data on the numerous SDOH constructs that inform health. They may also be limited in their ability to link to clinical data, such as data from the EHR due to a lack of available EHR data or technical challenges. Integrating SDOH data with clinical data – specifically, EHR data – requires common standards for collecting and communicating SDOH data [17]. This includes developing definitions for SDOH constructs and defining a geographic level for each construct. Some SDOH constructs are readily defined by their source (e.g., percent with a bachelor’s degree), while other constructs need to be derived (e.g., neighborhood poverty). Many constructs are available across geographic levels (e.g., census-based variables), while others need subject matter expertise (e.g., converting PM2.5 concentration based on grid size to census boundaries). SEED addresses these challenges by providing a data dictionary to define constructs and by providing FIPS codes at multiple geographic levels for variables.

There are some limitations to the SEED platform. Data on the prevalence of health conditions derived from DUHS, and Lincoln EHR data is limited to Durham County. DUHS and LCHC do not have sufficient coverage across other counties to provide reliable prevalence estimates. Collaborations with other health systems and adding national level data on the prevalence of health conditions from sources such as the CDC may address this in the future. SDOH data on the platform includes a curated list of variables that are commonly used. There are many more variables that can be added to the platform, including those suggested by community members. We plan to integrate application programming interfaces (APIs) to do this more efficiently. The sustainability of the platform is important. SEED is supported by the Duke Clinical and Translational Science Institute (CTSI), by investigators who include SEED in grant applications, and by pursuing grants that aim to democratize data for multiple end users. This supports effort for analysts and web developers so that data helpful for all users and data specific to projects, respectively, can be added to the website. The goal is to update existing data once a year and upload new data quarterly or semiannually.

## Conclusion

The SEED Health Atlas addresses many of the goals of the National Center for Advancing Translational Sciences by providing a promising tool for community members and translational researchers to use in their efforts to improve health equity [18]. SEED and tools like it are important because they allow for inferences on community health that can be translated into population-based interventions. They provide community stakeholders with information on community health that they act on to address social and environmental determinants of health. In addition, they facilitate the work of translational researchers who can review these data to develop new hypotheses or identify communities with specific needs for intervention. Looking ahead, SEED platform development will focus on incorporating broader geographic levels and additional SDOH constructs, and it will seamlessly link with EHR data using APIs. We will continue to solicit feedback from community members on potential new data and functionality to be added to the website. The goal is to expand SEED to promote awareness and dialog on community health needs and develop interventions to improve health equity.
